# A suite of software for processing MicroED data of extremely small protein crystals

**DOI:** 10.1107/S1600576714008073

**Published:** 2014-05-29

**Authors:** Matthew G. Iadanza, Tamir Gonen

**Affiliations:** a Janelia Farm Research Campus, Howard Hughes Medical Institute, 19700 Helix Drive, Ashburn, VA 20147, USA

**Keywords:** electron diffraction, structure determination, computer programs

## Abstract

Electron diffraction of extremely small three-dimensional crystals (MicroED) allows for structure determination from crystals orders of magnitude smaller than those used for X-ray crystallography. MicroED patterns, which are collected in a transmission electron microscope, were initially not amenable to indexing and intensity extraction by standard software, which necessitated the development of a suite of programs for data processing. The MicroED suite was developed to accomplish the tasks of unit-cell determination, indexing, background subtraction, intensity measurement and merging, resulting in data that can be carried forward to molecular replacement and structure determination. This *ad hoc* solution has been modified for more general use to provide a means for processing MicroED data until the technique can be fully implemented into existing crystallographic software packages. The suite is written in Python and the source code is available under a GNU General Public License.

## Abstract 2

Electron diffraction of extremely small three-dimensional crystals (MicroED) allows for structure determination from crystals orders of magnitude smaller than those used for X-ray crystallography. The MicroED suite was developed to accomplish the tasks of unit-cell determination, indexing, background subtraction, intensity measurement and merging, resulting in data that can be carried forward to molecular replacement and structure determination.

## 1. Introduction   

We recently presented the 2.9 Å resolution structure of hen egg white lysozyme determined by electron diffraction of three-dimensional microcyrstals 2 × 2 × 0.5 µm in size (Shi *et al.*, 2013 ▶). This technique, which we called MicroED, allows for structure determination from protein crystals that are more than six orders of magnitude smaller than those used for X-ray crystallography. In the future this may allow for protein structure determination for targets that have so far been unattainable.

Electrons provide an alternative to X-rays for diffraction studies of small protein crystals. The larger ratio of elastic to inelastic scattering coupled with the significantly smaller amount of energy deposited by inelastic electron scattering events (Henderson, 1995 ▶) increases the quantity of data that can be collected from small crystals before they are destroyed by radiation damage. This is tempered by the limited penetration of the electron beam compared to X-rays, which has generally restricted electron crystallography to very thin two-dimensional crystals (Abeyrathne *et al.*, 2011 ▶). Previous work to collect diffraction data from thin three-dimensional crystals found that crystals that were small enough to allow beam penetration were destroyed after the collection of only one or two diffraction patterns at the usual ∼20 e^−^ Å^−2^ dose rate (Glaeser, 1971 ▶). Therefore diffraction patterns had to be collected from multiple crystals, and several groups have been developing software for processing misoriented electron diffraction data (Jiang *et al.*, 2009 ▶, 2011 ▶) An analogous problem has been encountered and solved in the development of X-ray free-electron laser (XFEL)-based microcrystal diffraction methods (Chapman *et al.*, 2011 ▶; Boutet *et al.*, 2012 ▶).

MicroED data are collected using extremely low electron dose rate and under cryogenic conditions in a transmission electron microscope from crystals embedded in vitreous ice. A diffraction pattern is collected from a static crystal, which is then tilted to collect additional patterns at varying angles. Because the electron dose is very low, 0.01 e^−^ Å^−2^ s^−1^, multiple diffraction patterns (‘still diffraction’ tilt series) can be collected from a single crystal using a sensitive CMOS-based camera before significant radiation damage becomes apparent (Shi *et al.*, 2013 ▶). Related methods such as electron diffraction tomography and the diffraction rotation technique have been described before (Kolb *et al.*, 2007 ▶, 2008 ▶; Zhang *et al.*, 2010 ▶).

The electron diffraction data generated from MicroED are in principle no different than those from X-ray diffraction. Our attempts to index the diffraction patterns using *MOSFLM* (Leslie & Powell, 2007 ▶) resulted in errors, apparently due to a combination of the size of the Ewald sphere, inaccuracy in the tilt angles reported by the microscope compustage, and the fact that the pattern arises from static crystals while the program was expecting crystal oscillation. Such difficulties in processing electron diffraction data with *MOSFLM* have been reported by others (Nederlof *et al.*, 2013 ▶). However, some success was reported with *MOSFLM* when rotation electron diffraction was used, after the diffraction patterns had been centered and bad pixels removed. Other software, which was written specifically for processing electron diffraction data from patterns where the relational angle is known or not, is also available and includes *EDIFF* and *RED* (Jiang *et al.*, 2011 ▶; Wan *et al.*, 2013 ▶). Such software may have been useful in processing MicroED data, but we had difficulties in implementing the programs on our systems. Therefore, a suite of software was written to process the MicroED data for our initial proof of concept experiments, with the eventual goal of integration of these techniques into currently existing software such as *MOSFLM* (Leslie & Powell, 2007 ▶) or *CCTBX* (Adams *et al.*, 2010 ▶).

The MicroED suite contains eight programs that work together to accomplish essential data processing tasks: determination of the unit-cell size and orientation, spot prediction, indexing, and measuring spot intensities:

*Cataspot.py*: a graphical user interface (GUI) which allows the user to identify spots in diffraction patterns, record their *x*, *y* coordinates and prepare the data files used by subsequent programs.

*find_lengths*: rough determination of unit-cell lengths.

*calc_ucvectors*: rough determination of the vectors that describe the unit cell in reciprocal space.

*spot_index*: indexing of the spots for finer unit-cell vector determination.

*refine_spots*: refinement of the spots for unit-cell vector determination.

*UCR_index*: re-indexing of the images using the refined spots.

*recalculate_vectors*: calculation of more accurate unit-cell vectors from the new indexing.

*measure_intensities*: measurement of background-subtracted spot intensities.

The MicroED suite programs are designed to be cross-platform with any necessary modules, libraries and/or outside programs commonly available. All of the programs are implemented in Python 2.7 using the standard modules numpy (Oliphant, 2007 ▶) and Python Image Library (Secret Labs, 2013 ▶). The GUI requires Tkinter (Python Software Foundation, 2013 ▶), also a common python module. Two outside programs are required: *Gnuplot* (Williams & Kelley, 2011 ▶) and *ImageMagick* (ImageMagick, 2013 ▶), both of which are freely available. Any additional image processing can be performed with *FIJI* (Schindelin *et al.*, 2012 ▶).

## 2. Initial data processing   

The raw images used for data processing must be in tif format. This is a standard output option for *EM-MENU4 *(Ghadimi *et al.*, 2009 ▶), which was used for data collection in our initial work (Shi *et al.*, 2013 ▶). Other EM data formats can be converted into tif format using programs such as *em2em* (Image Science Software, 2013 ▶). In order to conserve memory, all of the illustrations drawn by the programs use a gif version of each image, which can be produced using *FIJI* or any other image manipulation program, the only requirement being that the gif version has the same name as the original file.

## 3. *Cataspot*   

**Figure 1:**
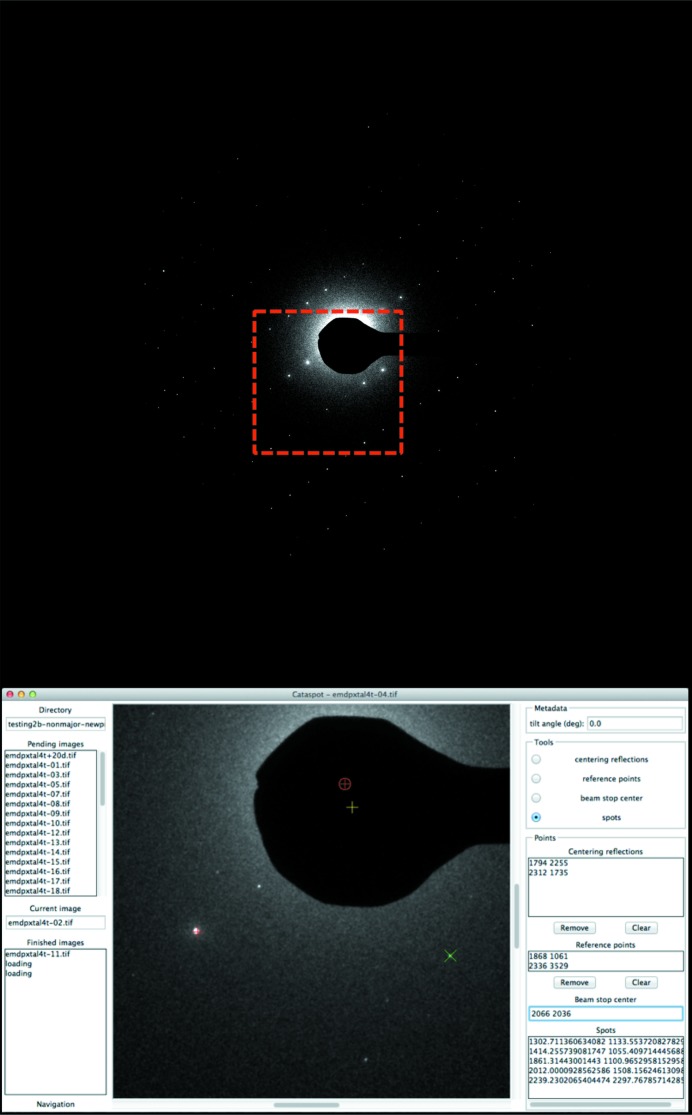
The *Cataspot* GUI. An electron diffraction pattern with the *Cataspot* GUI operating on the boxed region. Several user-selected spots including a centering spot (red plus), calculated beam center (red circled plus), beam stop center (yellow plus) and user chosen spot (green cross) are shown.

*Cataspot*’s GUI (Fig. 1 ▶) allows for batch processing of images, prompting the user to select points on the image and then determining relevant parameters and writing a data file used by the subsequent programs.

The first procedure performed by *Cataspot* is the determination of the beam center, which is calculated on the basis of one or more Freidel pairs selected by the user. The user is also able to specify the beamstop center, used later to calculate the beamstop mask. The program then allows the user to select additional spots to be used for unit-cell determination and reference spots which will be used to define the plane of the image for spot prediction.

## 4. Unit-cell determination   

The electron diffraction data processing suite *EDIFF* (Jiang *et al.*, 2011 ▶) provides a platform for determining unit-cell parameters from single electron diffraction patterns obtained from randomly oriented crystals. We encountered difficulties in implementing *EDIFF* on our systems, and inputting MicroED data into *EDIFF* caused unknown errors, which precluded us from using this program.

In our original paper, the structure determination of lysozyme was started with *a priori* knowledge of unit-cell dimensions and angles for the crystal (Cipriani *et al.*, 2012 ▶). Therefore a simplified unit-cell determination procedure was used only to verify the expected dimensions and angles.

*De novo* unit-cell determination using updated programs is now possible but will require significant trial and error on the part of the user. Techniques such as the Rossman Fourier analysis method used by *MOSFLM* (Powell, 1999 ▶; Steller *et al.*, 1997 ▶) or the ‘facet matching’ method of *EDIFF* would be much more effective for determination of an unknown unit cell. It is recommended to use the MicroED suite unit-cell determination programs as a last resort and to independently verify unit-cell dimensions using an outside program.

### 4.1. Determination of unit-cell lengths: *find_lengths*   

Unit-cell determination begins with the user picking 100–1000 spots from several diffraction patterns of various tilts. The user should attempt to choose a variety of spots so as to minimize the accidental selection of multiple appearances of a low-angle reflection over multiple patterns. A vector **v** is calculated for each spot, which defines its position relative to Cartesian coordinates (0, 0, 0):
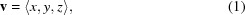


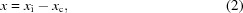


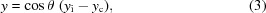


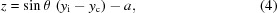
where *x*_i_ and *y*_i_ are the *x* and *y* coordinates on the diffraction pattern image, *x*_c_ and *y*_c_ are the *x* and *y* coordinates of the beam center, θ is the tilt angle, and *a* is a correction for the Ewald sphere curvature. Although Ewald sphere curvature also affects the *x* and *y* coordinates, it was determined that this difference was so small (∼1 pixel at 2.0 Å resolution) that it can be ignored. The effect of Ewald sphere curvature on the calculation of the *z* component of **v** is significant (∼12 pixels at 2.0 Å for our data set using our camera and microscope combination), so *a* is calculated as
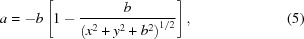


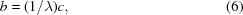
where λ is the electron wavelength, *x* and *y* are as calculated above, and *c* is the ångström to pixels conversion factor.

**Figure 2:**
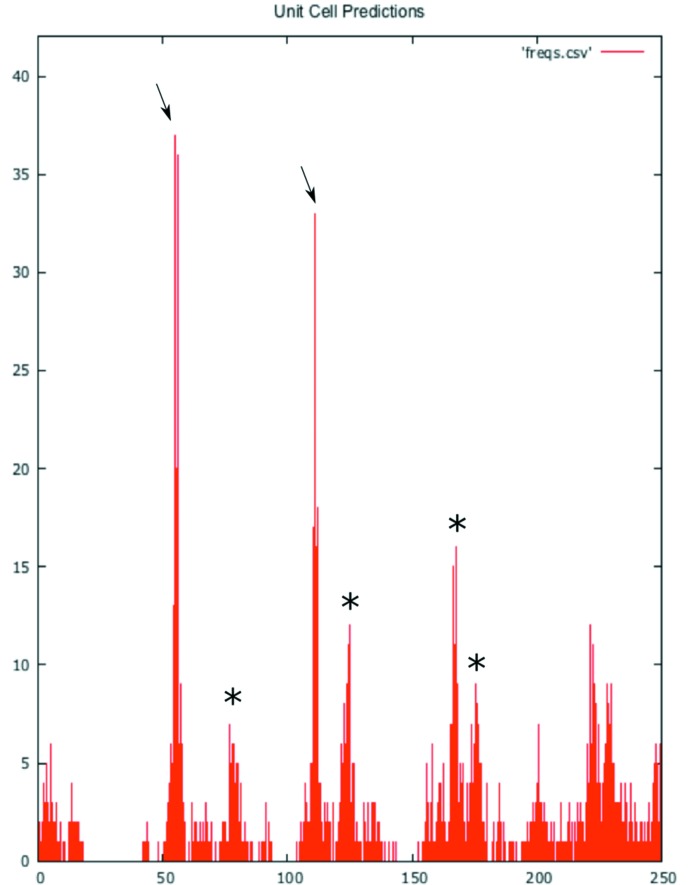
Unit-cell predictions for lysozyme by *1_find_lengths.py*. Predictions were made with approximately 900 spots chosen from 20 images over a −20 to 20° tilt. Peaks for the correct *a* and *b* (55 pixels) and *c* (112 pixels) unit-cell lengths are denoted with arrows. Peaks for *a* → 2*b* (65 pixels), 2(*a* → 2*b*) (130 pixels), *a* → 2*c* (156 pixels) and *a* → 3*b* (173 pixels) are also apparent (marked with stars).

After **v** is calculated for each spot, the distance between spots *d* is calculated for every pair of spots. For spots defined by **v**_*a*_ and **v**_*b*_, 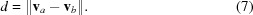
The unit-cell lengths can be estimated from the distribution of *d* for all spots. For two spots with adjacent Miller indices, *d* is equal to a unit-cell dimension. This distance cannot be smaller than the smallest unit-cell dimension, so the smallest peaks in the distribution of *d* represent the unit-cell dimensions. This process is not exact and still requires some user intuition. For example *d*_(000)(100)_ (between Miller indices 100 and 000) equals the *a* unit-cell dimension, but *d*_(110)(000)_ might be smaller than *d*_(000)(001)_ depending on the unit-cell dimensions. This, along with the possibility of multiple unit-cell dimensions having the same length, or one or more unit-cell lengths being close multiples of each other, means the user cannot simply pick the three shortest values of *d* as the unit-cell dimensions. The general formula for these cross-unit-cell vectors for a unit cell *a*, *b*, *c* with angles α, β, γ is
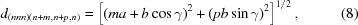
where *m* and *p* are integers. This allows for the calculation of the expected peaks in the distribution of *d*, which can be compared with the observed distribution and used to verify that the correct unit-cell dimensions have been chosen (Fig. 2 ▶). Observations of diffraction patterns that hit on or near major planes of the crystal also allows rough measurements of the unit-cell dimensions and angles directly from the patterns, which can be used to verify these findings.

### 4.2. Initial determination of unit-cell orientation: *calc_ucvectors*   

Once rough unit-cell dimensions have been determined, the orientation of the unit cell can be established. This is initially accomplished by using the spots that were chosen by the user. First the vectors **d** between all of the chosen spots are calculated:
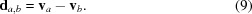
All of the vectors are compared with the three unit-cell dimensions and those within a user-specified threshold are kept. The remaining vectors are then compared with four reference vectors with Cartesian coordinates 〈1, 0, 0〉, (0, 1, 0〉, 〈0, 0, 1〉 and 〈1, 1, 0〉. The angle (σ) between the each vector and the reference vector is calculated by
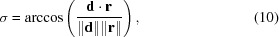
where **d** is the difference vector and **r** is the reference vector. This allows the vectors to be divided into roughly parallel groups based on the angles between the vector and the four reference vectors.

The orientations of the vectors in each group are determined by calculating the cross product of the vector and the 〈1, 0, 0〉 reference vector, and appropriate vectors are flipped, by multiplying by −1, so all vectors in each group are oriented in the same direction. The vectors in each group are averaged to produce a list of candidates for the unit-cell vectors. Each candidate is assigned a score based on the number of vectors that contributed to it. By examining the angles between the candidates and their scores, the correct unit-cell vectors can usually be chosen.

### 4.3. Refinement of the vectors: *spot_index*, *refine_spots*, *UCR_index* and *recalculate_vectors*   

Once the three vectors defining the unit cell have been chosen, they are used to predict spots on each image. The unit-cell vectors 〈
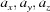
〉, 〈
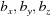
〉 and 〈
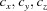
〉 are used to create a unit-cell matrix
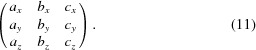
Two reference spots are chosen from each image. These spots are chosen because they have strong intensity and are thought to represent complete intensities where the Ewald sphere passed directly through the center of the spot. The *x*, *y*, *z* coordinates of the reference spots are calculated as above and their Miller indices determined by multiplying the *x*, *y*, *z* coordinates with the inverse unit-cell matrix:
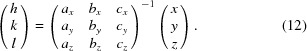
These values are rounded to the nearest integer, and a ‘check vector’ (**q**) normal to the plane containing the two reference points is calculated as
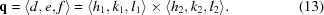
Every Miller index is then compared with the check vector. For any given Miller index *hkl*, the dot product of the Miller index and **q** can be used to determine if that Miller index lies on the same plane as the two reference spots.

**Figure 3:**
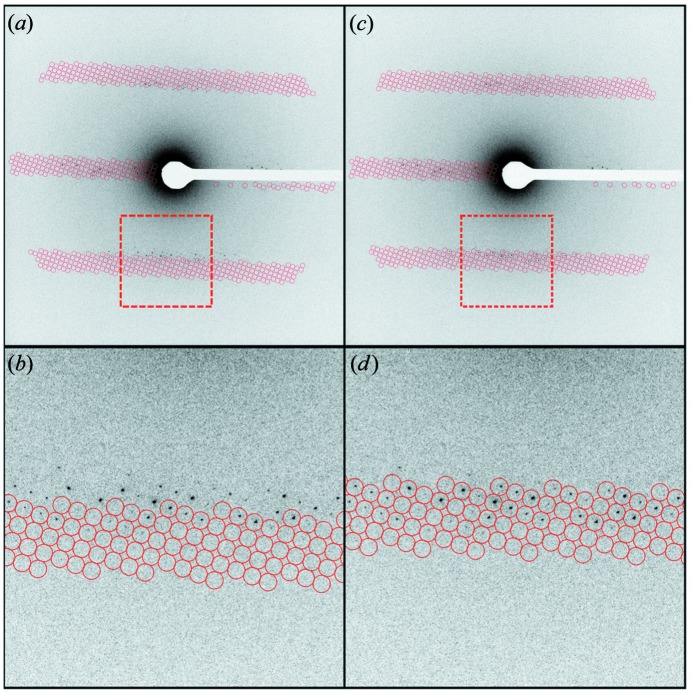
A demonstration of how the quality of reference points affects the accuracy of spot prediction. (*a*) A lysozyme diffraction pattern indexed with poor-quality reference points. (*b*) A zoomed-in view of the region of panel (*a*) bounded by the dashed line. (*c*) The same diffraction pattern indexed with higher-quality reference points. (*d*) A zoomed-in view of the region bounded by the dashed line in (*c*).

The two reference points are known to exist because they are visible on the diffraction pattern, so this can be used to predict the other spots that should appear on each diffraction pattern. The quality of the reference points chosen is critically important. The spots must be the user’s best estimation of Bragg peaks that were perfectly bisected by the Ewald sphere. A good rule of thumb is to choose spots that are of high relative intensity and have adjacent spots visible on both sides. Fig. 3 ▶ illustrates a diffraction pattern indexed with two different sets of reference points, demonstrating the effects of the reference set on the overall quality of the indexing.

**Figure 4:**
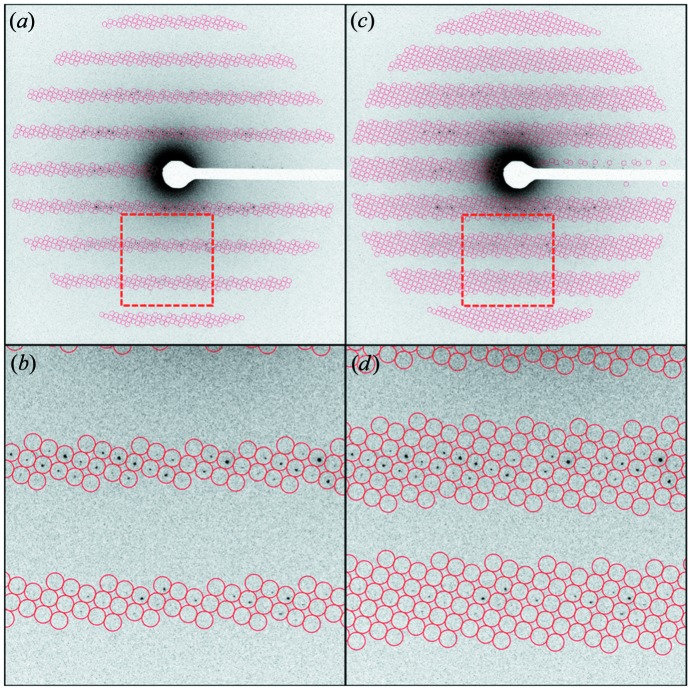
The effects of changing the Laue zone threshold on spot prediction. Predicted spots with 15% (*a*) and (*b*) and 30% (*c*) and (*d*) Laue zone thresholds drawn on a lysozyme diffraction pattern.

The probability that the dot product of any given Miller index and the check plane is exactly zero is very small. The calculation of the check plane is based on the locations of the reference spots, which introduces error as the measurement of these *x*, *y* coordinates will never be exactly perfect. To cope with this noise, the spots are instead compared with *L*; a ‘Laue zone threshold’, so named because its functional effect is to determine the widths of Laue zones in the spot predictions. Modifying the *L* value allows for compensation for inaccuracy in the tilt angle by expanding the size of the predicted Laue zones (Fig. 4 ▶). Raising this threshold results in the prediction of more spots but also increases the number of partial intensities recorded and ‘false positives’, indexing where no spot is actually observed.

After a list of spots has been created for each image, their *x*, *y*, *z* coordinates are calculated by
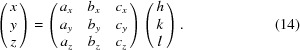
The *x*, *y*, *z* coordinates are then used to calculate the coordinates of the spot in two dimensions (*x*′, *y*′):
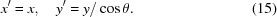
These coordinates are used to draw circles around the predicted spots on the diffraction patterns for visual inspection.

The initial spot predictions are dependent on the accuracy of the unit-cell vectors, which were determined from a limited set of points picked by the user. A second iteration of the vector finding process allows the refinement of the vectors for more accurate spot prediction.

The predicted spots are first refined by mass centering. A square box is drawn around each spot and the pixel values put in a matrix. Each row and column of the matrix is summed and the maximum pixel values of the rows and columns used to determine the actual center of mass for the spot. The box is moved to this center and the mass centering process repeated. If the second round of mass centering produces a large movement (more than one or two pixels in any given direction) the spot is discarded. This is to prevent the spot prediction from ‘walking’ between Miller indices.

After mass centering, the intensity of the spot is compared with the background intensity. A square and a circle where the circle diameter is equal to the square edge length are drawn, centered on the spot. The background intensity is defined as the mean pixel intensity of the area bounded by the square but outside the circle. The mean intensity of the area inside the circle is compared with the background intensity. Any spot with a low spot-to-background ratio is discarded. Because only intense spots that are cleanly bisected by the Ewald sphere are desired for unit-cell determination, this threshold is set high, usually around 10%.

The list of refined spots is then used to recalculate the unit-cell vectors. Because this list contains more spots and their locations are more accurate, the recalculated vectors produce better spot prediction and indexing. This process can be repeated iteratively until the unit-cell vectors are stable and accurate.

## 5. Indexing and intensity measurement: *measure_intensities*   

Once satisfactory unit-cell vectors have been obtained, the diffraction pattern image is indexed for a final time. The last set of spot indices is not mass centered. At this point the indexing should be accurate enough to capture all of the spots, and mass centering raises the risk of a spot ‘walking’ to an adjacent Miller index, which would lead to the intensity being attributed to the wrong reflection.

When the final indexing is complete the intensity of each spot is measured. The mean background is calculated for each spot as above and subtracted from each pixel within the circle, and the sum of the background-subtracted pixel values is recorded for that Miller index. The same mean spot intensity to background intensity comparison is then made as before, but a much lower threshold, usually ∼0.5%, is used to capture weak spots.

## 6. Merging: *p422_merge_maxonly* and *p422_merge_thresh*   

After all of the images have been indexed and the intensities extracted, intensity measurements from symmetry-related Miller indices must be merged. The symmetry relations of the different Miller indices are determined by the specific space group of the crystal. The proof of concept work took advantage of the *a priori* knowledge of the crystal space group. Without this information the space group must be determined by examining the unit-cell dimensions, angles and systematic absences using a tool such as *POINTLESS* (Evans, 2006 ▶). Merging programs were written specifically for *p*422 symmetries; merging data from other symmetries would require modification of the program.

**Figure 5:**
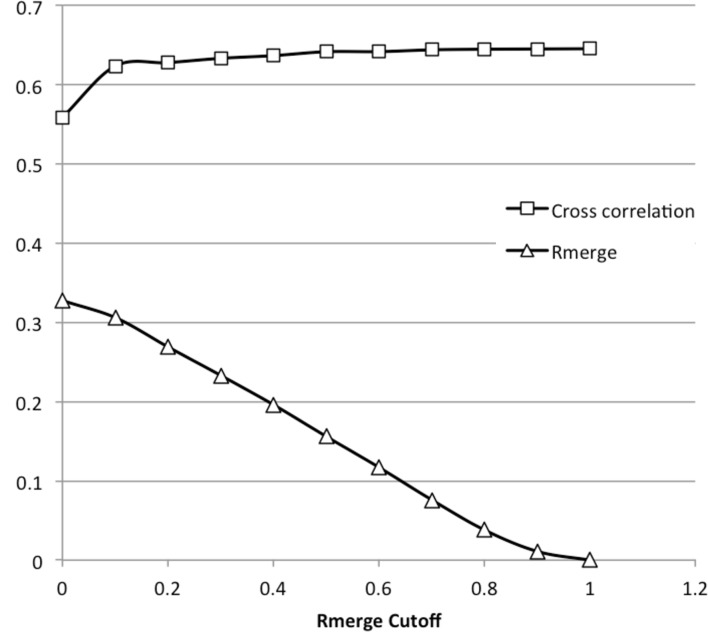
The effects of imposed *R*_merge_ cutoffs. The results of merging a lysozyme data set containing 36 823 intensity measurements with varying *R*_merge_ cutoffs, showing *R*_merge_ of the merged data set and its Pearson cross correlation to an X-ray diffraction data set collected from the same batch of crystals. In all cases the final merged data set contained 5460 intensity measurements.

Because our data originated from a static crystal (still shots), the probability of collecting partial reflections became much higher (Shi *et al.*, 2013 ▶). This led to inaccurate intensity measurements unless the partial reflections were scaled or excluded. To cope with this issue in our original work a strict cutoff was imposed. The program *p422_merge_maxonly* merges the data based on *p*422 symmetry. For any given reflection the largest recorded intensity was assumed to closely represent the complete reflection. Any measurements for that Miller index with smaller intensities were discarded. This is a crude method which precludes the calculation of *R*_merge_. Another program, *p422_merge_thresh*, allows the user to specify an *R*_merge_ cutoff: only spots within a specified range of the maximum recorded intensity for that Miller index are used for merging. Fig. 5 ▶ illustrates the effects of imposed cutoffs on the final *R*_merge_ and Pearson correlation coefficient for a lysozyme X-ray diffraction data set collected in-house. The full merged data set had an *R*_merge_ of 0.32 and 0.55 correlation to the X-ray data set. Overall the strict 1.0 cutoff (*i.e.* maximum measurements only) improved the cross correlation by approximately 10%, although most of this improvement was realized using a more permissive 0.1 cutoff.

The final output of the merging programs is a text file containing the Miller index, intensity, structure factor, SigI and SigF for each reflection. For intensity measurements originating from a single observation, SigI and SigF values cannot be calculated and they are instead estimated as the square root of the intensity and the square root of the structure factor, respectively. The output of the merging program can then be fed into the program *COMBAT* from the *CCP4* suite (Winn *et al.*, 2011 ▶) to generate an mtz file, which can be used for downstream applications.

## 7. Discussion   

This MicroED suite represents a refinement of an *ad hoc* software solution initially written for the determination of the structure of lysozyme by MicroED (Shi *et al.*, 2013 ▶). The programs were initially written in response to problems processing the data using currently available software and contain many workarounds resulting from logistical limitations that were described before as well as here. Although the programs have been modified for general use and now include a more user-friendly GUI they are not intended to be a mature suite for data processing. The final goal of this project is the integration of the MicroED techniques into currently available crystallography software. This should be concurrent with methodological improvements in MicroED.

## 8. Software availability   

All of the programs in the MicroED suite are available at http://www.github.com/gonenlab/2013UED.git.

## References

[bb1] Abeyrathne, P. D., Arheit, M., Kebbel, F., Castano-Diez, D., Goldie, K. N., Chami, M., Renault, L., Kühlbrandt, W. & Stahlberg, H. (2011). *Comprehensive Biophysics*, pp. 277–310. Waltham: Academic Press.

[bb2] Adams, P. D. *et al.* (2010). *Acta Cryst.* D**66**, 213–221.

[bb3] Boutet, S. *et al.* (2012). *Science*, **337**, 362–364.

[bb4] Chapman, H. N. *et al.* (2011). *Nature*, **470**, 73–77. 10.1038/nature09750 PMC3429598 21293373

[bb5] Cipriani, F., Röwer, M., Landret, C., Zander, U., Felisaz, F. & Márquez, J. A. (2012). *Acta Cryst.* D**68**, 1393–1399. 10.1107/S0907444912031459 22993093

[bb6] Evans, P. (2006). *Acta Cryst.* D**62**, 72–82. 10.1107/S0907444905036693 16369096

[bb7] Ghadimi, R., Daberkow, I., Sparlinek, P., Stumpf, M., Kofler, C. & Tietz, H. (2009). *MC2009*, Vol. 2, *Life Sciences*, edited by M. A. Pabst & G. Zellnig. Verlag der TU Graz.

[bb8] Glaeser, R. M. (1971). *J. Ultrastruct. Res.* **36**, 466–482. 10.1016/s0022-5320(71)80118-1 5107051

[bb9] Henderson, R. (1995). *Q. Rev. Biophys.* **28**, 171–193. 10.1017/s003358350000305x 7568675

[bb10] ImageMagick (2013). *ImageMagick: Convert, Edit, and Compose Images*, http://www.imagemagick.org/.

[bb11] Image Science Software (2013). *em2em: 3DEM Conversion Program.* Image Science Software GmbH, Berlin, Germany, http://www.imagescience.de/em2em.html.

[bb12] Jiang, L., Georgieva, D. & Abrahams, J. P. (2011). *J. Appl. Cryst.* **44**, 1132–1136.

[bb13] Jiang, L., Georgieva, D., IJspeert, K. & Abrahams, J. P. (2009). *CISP ’09. 2nd International Congress on Image and Signal Processing*, 17–19 October 2009, pp. 1–5. Red Hook: Curran Associates.

[bb14] Kolb, U., Gorelik, T., Kübel, C., Otten, M. T. & Hubert, D. (2007). *Ultramicroscopy*, **107**, 507–513. 10.1016/j.ultramic.2006.10.007 17234347

[bb15] Kolb, U., Gorelik, T. & Otten, M. T. (2008). *Ultramicroscopy*, **108**, 763–772. 10.1016/j.ultramic.2007.12.002 18282662

[bb16] Leslie, A. G. W. & Powell, H. R. (2007). *Evolving Methods for Macromolecular Crystallography*, edited by R. J. Read & J. L. Sussman, pp. 41–51. Dordrecht: Springer.

[bb17] Nederlof, I., van Genderen, E., Li, Y.-W. & Abrahams, J. P. (2013). *Acta Cryst.* D**69**, 1223–1230. 10.1107/S0907444913009700 PMC3689525 23793148

[bb18] Oliphant, T. E. (2007). *Comput. Sci. Eng.* **9**, 10–20.

[bb19] Powell, H. R. (1999). *Acta Cryst.* D**55**, 1690–1695. 10.1107/s0907444999009506 10531518

[bb20] Python Software Foundation (2013). *Tkinter – Python Interface to Tcl/Tk*, http://docs.python.org/2/library/tkinter.html.

[bb21] Schindelin, J. *et al.* (2012). *Nat. Methods*, **9**, 676–682. 10.1038/nmeth.2019 PMC3855844 22743772

[bb22] Secret Labs (2013). *Python Imaging Library (PIL)*, Secret Labs AB, Linköping, Sweden, http://www.pythonware.com/products/pil/.

[bb23] Shi, D., Nannenga, B. L., Iadanza, M. G. & Gonen, T. (2013). *eLife*, **2**, e01345. 10.7554/eLife.01345 PMC3831942 24252878

[bb24] Steller, I., Bolotovsky, R. & Rossmann, M. G. (1997). *J. Appl. Cryst.* **30**, 1036–1040.

[bb25] Wan, W., Sun, J., Su, J., Hovmöller, S. & Zou, X. (2013). *J. Appl. Cryst.* **46**, 1863–1873. 10.1107/S0021889813027714 PMC3831301 24282334

[bb26] Williams, T. & Kelley, C. (2011). *Gnuplot 4.5*, http://gnuplot.info.

[bb27] Winn, M. D. *et al.* (2011). *Acta Cryst.* D**67**, 235–242.

[bb28] Zhang, D., Oleynikov, P., Hovmoller, S. & Zou, Z. (2010). *Z. Kristallogr.* **225**, 94–102.

